# Effect of *Bouvardia ternifolia* Root Extract on Brain Structures, Oxidative Stress, and p53 Expression in a Rat Model of Cerebral Ischemia/Reperfusion

**DOI:** 10.3390/ph18111678

**Published:** 2025-11-05

**Authors:** Yury Maritza Zapata-Lopera, Gabriela Trejo-Tapia, Edgar Cano-Europa, Vanessa Blas-Valdivia, Maribel Herera-Ruiz, Francisco A. Miguel-Martínez, Enrique Jiménez-Ferrer

**Affiliations:** 1 Centro de Investigación Biomédica del Sur, Instituto Mexicano del Seguro Social, Xochitepec 62790, Mexico; yzapatal1600@alumno.ipn.mx (Y.M.Z.-L.);; 2 Centro de Desarrollo de Productos Bióticos, Instituto Politécnico Nacional, Yautepec 62730, Mexico; 3 Laboratorio de Metabolismo I, Departamento de Fisiología, Escuela Nacional de Ciencias Biológicas, Instituto Politécnico Nacional, Ciudad de México 07738, Mexico; edgarcanoeuropa@yahoo.com.mx (E.C.-E.); vaneblasval@yahoo.com.mx (V.B.-V.); franciscoa.mm0399@gmail.com (F.A.M.-M.)

**Keywords:** *Bouvardia ternifolia*, cerebral ischemia/reperfusion, hippocampus, oxidative stress, p53 protein

## Abstract

**Background/Objectives:** Cerebral ischemia and reperfusion injury, induced by bilateral common carotid artery occlusion and reperfusion (BCCAO/R), cause extensive neuronal damage and cognitive impairment. *Bouvardia ternifolia* (BtD), a plant known for its anti-inflammatory and neuroprotective effects, may offer therapeutic benefits against ischemic injury. This study aimed to evaluate the neuroprotective effects of BtD root extract on neuronal integrity, oxidative stress, and p53 protein expression following global cerebral ischemia in rats. **Methods:** Adult male Sprague Dawley rats were subjected to the BCCAO/R procedure for 60 min, followed by six days of reperfusion. Experimental groups included BCCAO/R+BtD, BCCAO/R+silymarin (reference control), BCCAO/R+vehicle, and sham controls. Neuronal morphology in the cortex, striatum, hippocampus, and cerebellum was assessed histologically. Oxidative stress markers, including reactive oxygen species (ROS), lipid peroxidation (LPO), reduced glutathione (GSH), and superoxide dismutase (SOD), were measured, along with the expression of p53 protein. **Results:** Treatment with BtD significantly decreased oxidative stress markers—LPO (82.2%), ROS (88.2%), GSH (66.5%), and SOD (54%)—and reduced p53 expression levels by 75%. Histological evaluation revealed that neurons in the BCCAO/R+BtD and BCCAO/R+silymarin groups maintained normal morphology, characterized by elongated cells and well-defined nuclei. In contrast, the BCCAO/R+vehicle group exhibited marked neuronal damage, including pyknosis, nuclear fragmentation, and interstitial edema, particularly in the hippocampal CA1 and cortical regions. BtD treatment significantly preserved neuronal structure and enhanced antioxidant defenses. **Conclusions:** *Bouvardia ternifolia* extract demonstrates neuroprotective potential in cerebral ischemia by maintaining neuronal architecture, reducing oxidative stress, and modulating p53 expression, supporting its therapeutic relevance in ischemia–reperfusion injury.

## 1. Introduction

Cerebral ischemia/reperfusion (BCCAO/R) is a cerebrovascular condition characterized by a transient reduction in cerebral blood flow, leading to selective neuronal loss in vulnerable brain regions such as the hippocampus, neocortex, and striatum. In gerbil models, as little as five minutes of forebrain ischemia/reperfusion induces neuronal death in the cornu ammonis 1 (CA1) subfield of the hippocampus, often resulting in impairments in spatial memory and learning. Within 4 to 5 days post-ischemia, neuronal damage may also extend to the CA2 and CA3 subfields [1]. Clinically, cerebral BCCAO/R is associated with neurodegeneration, cognitive dysfunction, memory deficits, and, in progressive cases, the development of dementia [2]. Oxidative stress plays a central role in the pathophysiology of cerebral BCCAO/R, leading to the accumulation of reactive oxygen species (ROS) and subsequent damage to cellular macromolecules, including DNA, proteins, and lipids. In addition, the ischemic cascade is marked by an overproduction of proinflammatory mediators that exacerbate neuronal injury [3].

In cerebral ischemia, the deprivation of oxygen and glucose leads to increased calcium influx into mitochondria, resulting in elevated production of reactive oxygen species (ROS) and the release of cytochrome c. These events activate caspase cascades, ultimately triggering apoptosis [4]. The brain’s primary defense against ROS and lipid peroxidation (LPO) is its endogenous antioxidant system, which involves key enzymes such as superoxide dismutase (SOD), catalase (CAT), and glutathione peroxidase (GPx), working collectively to neutralize oxidative damage [5].

The p53 protein plays a pivotal role in cellular homeostasis, regulating cell cycle progression, DNA repair, and apoptosis. Different isoforms of p53 have been shown to influence neuronal fate following ischemic injury. The p53-arginine variant promotes neuronal apoptosis, whereas the p53-proline variant contributes to neuronal repair. This reparative mechanism involves the recruitment of endothelial progenitor cells from the bone marrow to the site of cerebral injury [6,7]. Puerarin, an antioxidant compound extracted from the root of *Pueraria lobata*, has been widely used as an effective therapeutic agent for cerebral ischemia/reperfusion injury. Puerarin physically binds to p53, inhibiting its Ser15 phosphorylation and preventing p53 activation, which consequently leads to the inhibition of ferroptosis [8].

Numerous studies suggest that diets rich in antioxidants, including flavonoids and plant-derived polyunsaturated fatty acids, may contribute to the prevention of neurodegenerative disease [9]. Honokiol and plumbagin inhibit the NADPH oxidase (NOX) enzyme [10], while allopurinol, a xanthine oxidase (XO) inhibitor, reduces infarct volume by 32–35%, decreases cerebral edema formation, and improves blood–brain barrier integrity [11].

Numerous bioactive compounds have been identified as inhibitors of myeloperoxidase (MPO) activity and can help alleviate cerebral ischemia–reperfusion injury. This group includes bioflavonoids such as quercetin, rutin, eriodictyol, isorhamnetin, biochanin A, and baicalin; polyphenols such as resveratrol, curcumin, and cannabidiol; as well as alkaloids, saponins, terpenoids, and coumarins [12].

Resveratrol scavenges free radicals, modulates signaling pathways such as SIRT1 and Nrf2, and inhibits inflammatory mediators like NF-*κ*B [13]. In a rat model of cerebral BCCAO/R, quercetin delivered after BCCAO/R injury via i.p. injection (30–60 mg/kg) enhances neuronal survival by promoting M2 polarization of microglia/macrophages and modulating the phosphatidylinositol 3-kinase (PI3K)/protein kinase B (Akt)/NF-*κ*B axis [14].

The transcription factor nuclear factor erythroid 2–related factor 2 (Nrf2) plays a key role in mitigating oxidative stress during ischemia by regulating signaling pathways involving Keap1 proteins, PI3K/AKT, MAPK, nuclear factor kappa B (NF-κB), and heme oxygenase-1 (HO-1). Excessive ROS production following cerebral ischemia activates Nrf2, which in turn induces the transcription of various antioxidant genes, thereby reducing blood–brain barrier disruption and inflammation [15].

Rutaecarpine improves neuronal damage and inhibits apoptosis, inflammation, and oxidative stress by regulating the ERK1/2 and Nrf2/HO-1 signaling pathways in rats with cerebral ischemia–reperfusion injury [16]. Theaflavin alleviates cerebral ischemia–reperfusion injury by reversing miRNA1283-mediated inhibition of Nrf2 and reducing oxidative stress [17]. Carvacryl acetate provides neuroprotection through activation of the Nrf2 signaling pathway [18]. Geraniin protects against cerebral ischemia–reperfusion injury by suppressing oxidative stress and neuronal apoptosis via regulation of the Nrf2/HO-1 pathway [19]. Vitexin may enhance Akt and eNOS phosphorylation to maintain blood–brain barrier (BBB) integrity in an ischemic stroke model [20].

*Bouvardia ternifolia*, a Mexican species from the Rubiaceae family, has been traditionally used in various medicinal systems, including traditional Chinese and Mexican medicine, for its therapeutic potential. Its roots and rhizomes possess antioxidants, anti-ischemic, and antithrombotic properties [3] and have been used to treat conditions such as tuberculosis, menoxenia, rheumatism, contusions, hematemesis, anemia, and lipomas [21]. In traditional Mexican medicine, the root is applied to chronic wounds, and recent pharmacological studies have confirmed its anti-inflammatory effects, particularly through inhibition of the NF-κB pathway in the RAW 264.7 macrophage cell line [22]. Additionally, the aerial parts of *B. ternifolia* have been used for treating dysentery, rabies, hematemesis, and heart heat, and have demonstrated antitussive and stimulant properties (Digital Library of Traditional Mexican Medicine). Chemical studies carried out on the methanolic extract of the leaves, flowers, and stems of the species have revealed the presence of three cyclic hexapeptides: bouvardin, deoxy-bouvardin, and 6-O-methylbouvardin [23,24]; The presence of triterpenic acids, such as ursolic acid and oleanolic acid, has been reported in the hexanic and methanolic extracts of the roots, as well as in the chloroformic extract of the stem [25]. 3-O-quercetin glucopyranoside, rutin, ursolic and oleanolic acid, 3-O-quercetin rhamnopyranoside, chlorogenic acid, and scopoletin have been reported in the aerial parts [26]. Bouvardin, scopoletin, ternifolial, and ternifoliol have been reported in the root [27]. Nine compounds were identified in the dichloromethane (BtD) root extract: ternifolial, rubiyunnanin H, (M) lup-20(29)-en-3-ol-acetate (3β), (M) D, α-tocopherol, (R) squalene, (M) 1H-inden-1-one, 5-(1,1-dimethyl-ethyl)-2,3-dihydro-3,3-dimethyl-, (M) 2-nonadecanone, (M) s-indacene-1,7-dione, 2,3,5,6-tetrahydro-3,3,5,5-tetramethyl-, and (M) 3-carene [28]. Accordingly, this study aimed to investigate the neuroprotective effects of *Bouvardia ternifolia* root extract (BtD), with a particular focus on its impact on oxidative stress biomarkers, p53 expression, and histological changes in neuronal tissue following cerebral ischemia/reperfusion

## 2. Results

### 2.1. Effect of BtD Extract on BCCAO/R-Induced Oxidative Stress and Changes in the Brain’s Redox Environment

**Figure 1:**
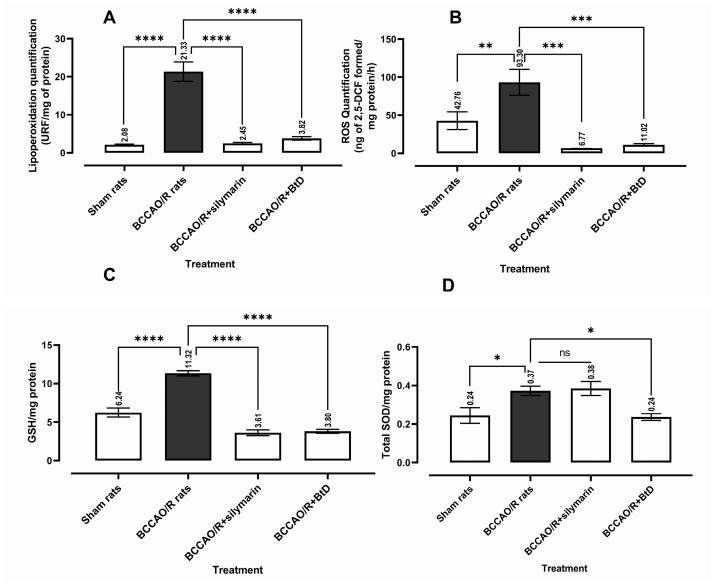
Effect of *Bouvardia ternifolia* (BtD) extract (300 mg/kg) on BCCAO/R-induced oxidative stress and changes in the brain’s redox environment. Oxidative stress markers: reactive oxygen species (ROS), lipid peroxidation (LPO) (**A**,**B**). Antioxidant enzyme activity reduced glutathione (GSH), superoxide dismutase (SOD) (**C**,**D**). RFU, relative fluorescence units. Data are presented as mean ± SEM. Variables were assessed using one-way analysis of variance (ANOVA) with Dunnett’s post test. (*) *p* < 0.05, (**) *p* < 0.01, (***) *p* < 0.001, (****) *p* < 0.0001, compared to the BCCAO/R group. (*n* = 7 per group). No significant difference (ns).

Figure 1A presents the quantitative analysis of reactive oxygen species (ROS) levels. The Sham group exhibited significantly lower ROS levels compared to the BCCAO/R damage group (Mean ± SEM 42.76, *p* < 0.0088). Treatment with silymarin (BCCAO/R+silymarin) resulted in a marked reduction in ROS levels (Mean ± SEM 6.767, *p* < 0.0001), while treatment with *Bouvardia ternifolia* extract (BCCAO/R+BtD) also significantly decreased ROS generation (Mean ± SEM 11.02, *p* < 0.0003) compared to the BCCAO/R group. A one-way ANOVA confirmed a significant difference among the groups (F(3,15) = 13.23, *n* = 7, *p* < 0.0002). The quantitative analysis of oxidative stress markers indicates that BCCAO/R induces significant oxidative damage. ROS levels were markedly elevated in the BCCAO/R group compared to Sham, confirming the expected overproduction of reactive oxygen species following ischemic injury. Treatment with *Bouvardia ternifolia* BtD extract or silymarin significantly reduced ROS generation, demonstrating their potent antioxidant effects in mitigating oxidative stress.

Figure 1B shows the levels of lipid peroxidation (LPO). The BCCAO/R group displayed elevated LPO levels (Mean ± SEM 21.33) compared to the Sham group (Mean ± SEM 2.084, *p* < 0.0001). Silymarin treatment significantly reduced LPO (Mean ± SEM 2.451, *p* < 0.0001), as did BtD treatment (Mean ± SEM 3.818, *p* < 0.0001) when compared to the BCCAO/R group. The one-way ANOVA confirmed a significant group effect (F(3,13) = 58.27, *n* = 6, *p* < 0.0001). Similarly, LPO, a marker of membrane oxidative damage, was significantly higher in the BCCAO/R group and markedly decreased following BtD or silymarin treatment. This suggests that BtD protects neuronal membranes from peroxidative damage, comparable to the known neuroprotective activity of silymarin.

Figure 1C illustrates the effects of treatments on reduced glutathione (GSH) levels. The BCCAO/R group had the highest GSH levels (Mean ± SEM 11.32). In contrast, the Sham (Mean ± SEM 6.236), BCCAO/R+silymarin (mean = 3.611), and BCCAO/R+BtD (mean = 3.801) groups all showed significantly lower GSH levels (*p* < 0.0001 for each comparison with BCCAO/R). The one-way ANOVA revealed a significant difference among groups (F (3,18) = 73.4, *n* = 6, *p* < 0.0001). The observed increase in GSH levels following BCCAO/R may appear counterintuitive, as oxidative stress typically depletes glutathione. This elevation could reflect a compensatory upregulation of endogenous antioxidant defenses in response to acute oxidative insult. Previous studies have reported transient increases in GSH or related thiol antioxidants during the early reperfusion phase, likely as an adaptive mechanism to counteract ROS accumulation. Thus, the data may represent a protective, homeostatic response rather than a paradoxical effect, highlighting the dynamic regulation of redox balance following cerebral ischemia/reperfusion

Figure 1D depicts superoxide dismutase (SOD) activity. The BCCAO/R group had a mean activity of 0.3726, while the BCCAO/R+silymarin group showed a slightly higher mean (0.3843), which was not significantly different (*p* > 0.05). However, both the Sham (Mean ± SEM 0.2441, *p* < 0.0180) and BCCAO/R+BtD (Mean ± SEM 0.2362, *p* < 0.0124) exhibited significantly lower SOD activity compared to BCCAO/R. One-way ANOVA results indicated a statistically significant difference among groups (F(3,13) = 6.945, *n* = 6, *p* = 0.049). Regarding SOD activity, BCCAO/R increased enzyme activity compared to Sham, possibly as an adaptive response to elevated superoxide production. BtD treatment reduced SOD activity toward baseline levels, consistent with its ROS-scavenging capacity, while silymarin did not significantly alter SOD compared to BCCAO/R.

Collectively, these findings indicate that BtD effectively attenuates oxidative stress by reducing ROS and lipid peroxidation and restoring antioxidant homeostasis, supporting its neuroprotective potential in ischemia/reperfusion injury.

### 2.2. BtD Regulates p53 Protein Expression

**Figure 2:**
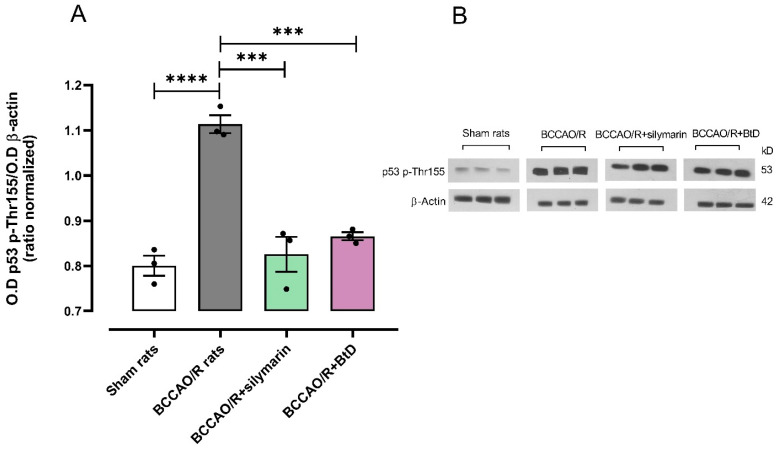
Effect of *Bouvardia ternifolia* (BtD) extract (300 mg/kg) on p53 protein expression following cerebral ischemia/reperfusion (BCCAO/R). (**A**) Quantification of p53 protein expression by optical density (O.D.). (**B**) Representative Western blot showing p53 levels across experimental groups, illustrating the impact of BtD extract on BCCAO/R-induced brain stress via the p53 pathway. Data are presented as mean ± SEM. Statistical analysis was performed using one-way ANOVA followed by Dunnett’s post hoc test. *** *p* < 0.001, **** *p* < 0.0001 vs. BCCAO/R group. (*n* = 7 per group).

Figure 2A,B show the effects of *Bouvardia ternifolia* extract (BtD, 300 mg/kg) and silymarin (50 mg/kg) on p53 protein expression in rats subjected to cerebral ischemia/reperfusion (BCCAO/R). The BCCAO/R damage control group exhibited a significant increase in p53 protein levels, reaching 111% relative to the Sham group (mean = 1.11), indicative of elevated pro-apoptotic signaling (*** *p* < 0.0001). Treatment with BtD extract (BCCAO/R+BtD) significantly reduced p53 expression by 75% (mean = 0.86, *p* < 0.0003), while silymarin treatment (BCCAO/R+silymarin) resulted in a 71% reduction (mean = 0.82, *p* < 0.0001). These results demonstrate that both BtD and silymarin effectively downregulated p53 protein expression, restoring levels closer to those observed in the Sham group and suggesting a protective effect against apoptosis in ischemic brain tissue.

### 2.3. Effect of BtD Extract on Neuronal Morphology at 6 Days of Reperfusion

#### 2.3.1. Cerebral Cortex

**Figure 3:**
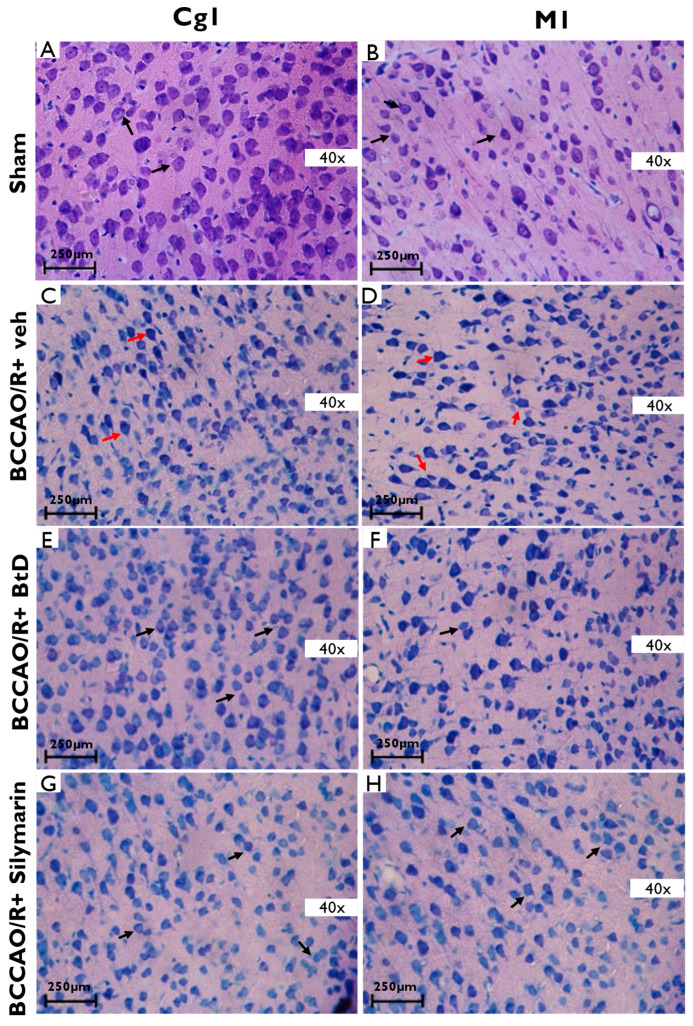
Representative photomicrographs of cortical regions 40× from rats subjected to cerebral ischemia/reperfusion (BCCAO/R) and treated with *Bouvardia ternifolia* dichloromethane extract (BtD, 300 mg/kg). (**A**) Sham Cg1; (**B**) Sham M1; (**C**) images show the anterior cingulate cortex (Cg1) and (**D**) primary motor cortex (M1). In the BCCAO/R+veh group (**C**,**D**) induced pronounced neuronal damage, including cell death, nuclear deformation (pyknosis and karyorrhexis), and interstitial edema (red arrows). In contrast, these histopathological alterations were markedly reduced in rats treated with BCCAO/R+BtD extract (**E**,**F**) or BCCAO/R+Silymarin (**G**,**H**) (black arrows), indicating preservation of cortical architecture. Tissue sections were stained with toluidine blue. Scale bar: 250 µm.

Figure 3 illustrates the histopathological changes observed in the cerebral cortex—specifically the anterior cingulate cortex (Cg1) and primary motor cortex (M1)—following cerebral ischemia/reperfusion (BCCAO/R). In the Sham group (Figure 3A,B), cortical neurons displayed normal morphology with well-defined membranes, centrally located nuclei, and preserved cytoarchitecture. In contrast, the BCCAO/R+veh damage control group (Figure 3C,D) showed pronounced pathological features, including pyknosis (irreversible chromatin condensation), karyorrhexis (nuclear fragmentation), atrophic necrotic neurons, eccentric nuclei, interstitial edema, and disrupted cortical cytoarchitecture. Treatment with *Bouvardia ternifolia* extract (BCCAO/R+BtD; Figure 3E,F) and silymarin (BCCAO/R+silymarin; Figure 3G,H) markedly attenuated these histological abnormalities. Both treatments preserved neuronal structure and reduced signs of ischemic damage when compared to the untreated BCCAO/R group, indicating neuroprotective effects in the cortex.

#### 2.3.2. Cerebral Striatum

**Figure 4:**
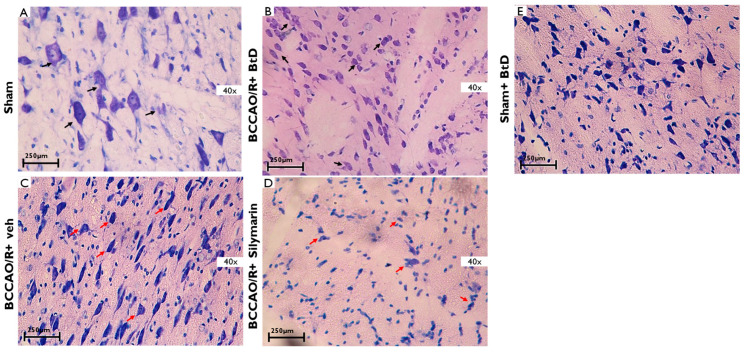
Representative photomicrographs of striatum regions 40× from rats subjected to cerebral ischemia/reperfusion (BCCAO/R) and treated with *Bouvardia ternifolia* BtD extract (300 mg/kg). Ischemia/reperfusion leads to cell death, deformed nuclei, and edema (red arrows). (**A**) Sham; (**B**) BCCAO/R+BtD; (**C**) BCCAO/R+veh; (**D**); BCCAO/R+Silymarin; (**E**) Sham+BtD. In contrast, these histopathological alterations were markedly reduced in rats treated with BCCAO/R+BtD extract (**B**) or BCCAO/R+silymarin (**D**) (black arrows), indicating preservation of cortical architecture. Tissue sections were stained with toluidine blue. Scale bar: 250 µm.

Figure 4 illustrates histological changes in the striatum following cerebral ischemia/reperfusion (BCCAO/R) and the effects of treatment with *Bouvardia ternifolia* extract (BtD) or silymarin. In the Sham group (Figure 4A), striatal neurons exhibited normal morphology, with elongated cell bodies and clearly defined, centrally located nuclei. In contrast, the BCCAO/R+veh damage group (Figure 4C) showed prominent histopathological alterations, including pyknosis, irregular cell shapes, and loss of nuclear definition (red arrows), consistent with ischemic injury. Treatment with BtD extract (BCCAO/R+BtD; Figure 4B) preserved striatal cell morphology, showing neurons with well-defined nuclei and elongated cytoplasm bordered by intact membranes—like those in the Sham group. However, in the silymarin-treated group (BCCAO/R+silymarin; Figure 4D), neuronal cells appeared smaller and more irregular, with increased hyperpigmentation and poorly defined nuclei (red arrows), suggesting less protection compared to BtD.

#### 2.3.3. Cerebral Hippocampus

**Figure 5:**
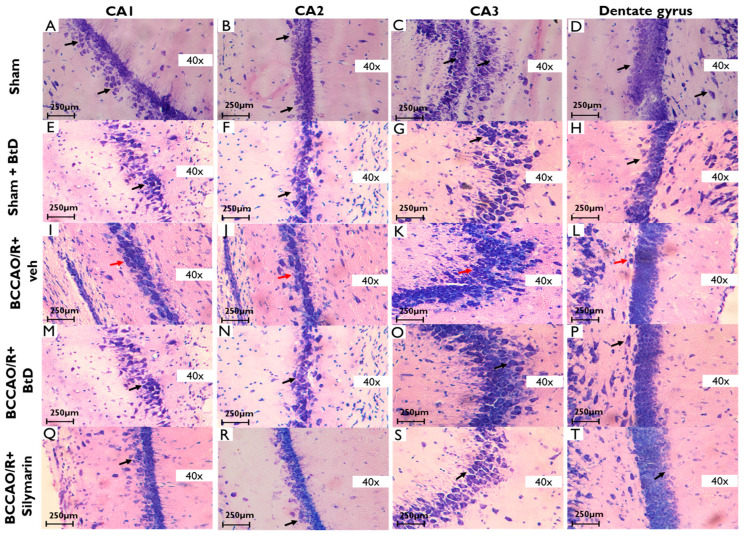
Representative photomicrographs of the hippocampus 40× (CA1, CA2, CA3, and dentate gyrus) regions from rats subjected to cerebral ischemia/reperfusion (BCCAO/R) and treated with *Bouvardia ternifolia* BtD extract (300 mg/kg). (**A**) Sham CA1; (**B**) Sham CA2; (**C**) Sham CA3; (**D**) Sham dentate gyrus; (**E**) Sham+BtD CA1; (**F**) Sham+BtD CA2; (**G**) Sham+BtD CA3; (**H**) Sham+BtD dentate gyrus; (**I**) BCCAO/R+veh CA1; (**J**) BCCAO/R+veh CA2; (**K**) BCCAO/R+veh CA3; (**L**) BCCAO/R+veh dentate gyrus; (**M**) BCCAO/R+BtD CA1; (**N**) BCCAO/R+BtD CA2; (**O**) BCCAO/R+BtD CA3; (**P**) BCCAO/R+BtD dentate gyrus; (**Q**) BCCAO/R+Silymarin CA1; (**R**) BCCAO/R+Silymarin CA2; (**S**) BCCAO/R+Silymarin CA3; (**T**) BCCAO/R+Silymarin dentate gyrus. Sham+BtD Ischemia/reperfusion results in cell death, deformed nuclei, and edema (red arrows). In contrast, these histopathological alterations were markedly reduced in rats treated with BCCAO/R+BtD extract or silymarin (black arrows), indicating preservation of cortical architecture. Tissue sections were stained with toluidine blue. Scale bar: 250 µm.

Figure 5 presents histological evaluation of hippocampal regions CA2 and CA3 following ischemia/reperfusion injury and treatment with *Bouvardia ternifolia* extract (BtD) or silymarin. In the Sham group, pyramidal neurons in CA2 and CA3 regions displayed normal architecture, with large, round, and well-defined nuclei, abundant cytoplasm, and organized laminar cytoarchitecture, including clearly distinguishable pyramidal, radiated, and lacunosum-molecular layers (Figure 5B,C). No evidence of interstitial edema or cellular disarray was observed. In contrast, the BCCAO/R+veh group exhibited marked structural degeneration (Figure 5L,M), including loss of pyramidal cell organization, nuclear condensation, signs of inflammation, and evidence of oxidative stress. Neurons showed pyknosis, shrinkage, and disrupted cytoarchitecture, consistent with ischemic injury. Following treatment with BtD extract (BCCAO/R+BtD; Figure 5O), hippocampal neurons showed notable improvement. Many CA3 neurons retained structural integrity and visible nuclei; however, some cells appeared smaller, loosely arranged, and exhibited pyknotic nuclei—indicating partial neuroprotection. In the BCCAO/R+silymarin group, histological appearance closely resembled that of the Sham group. Pyramidal neurons had clearly defined nuclei and cytoplasm, and no irregular, hyperpigmented, or necrotic cells were observed—suggesting a strong neuroprotective effect.

#### 2.3.4. Cerebellum

**Figure 6:**
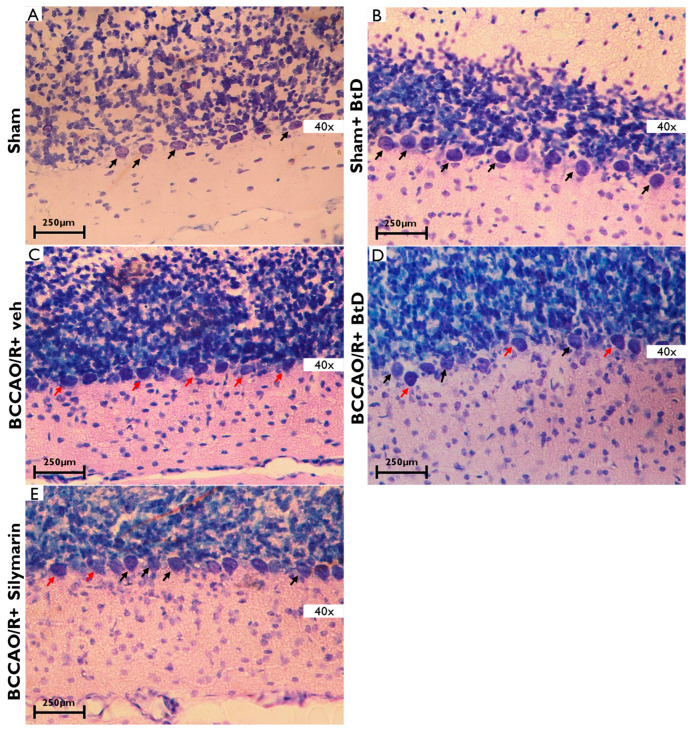
Representative photomicrographs of cerebellum regions 40× from rats subjected to cerebral ischemia/reperfusion (BCCAO/R) and treated with *Bouvardia ternifolia* BtD extract (300 mg/kg). (**A**) Sham; (**B**) Sham+BtD; (**C**) BCCAO/R+BtD; (**D**) BCCAO/R+veh; (**E**) BCCAO/R+Silymarin. Ischemia/reperfusion induces cell death, deformed nuclei, and edema (red arrows). In contrast, these histopathological alterations were markedly reduced in rats treated with BCCAO/R+BtD extract or silymarin (black arrows), indicating preservation of cortical architecture. Tissue sections were stained with toluidine blue. Scale bar: 250 µm.

Figure 6 illustrates the histological features of the cerebellar cortex, emphasizing the Purkinje cell layer and adjacent granular layer, across experimental groups. In the Sham group (Figure 6A), Purkinje cells appeared round with large, centrally located nuclei and were organized in a well-defined monolayer between the molecular and granular layers. A similar morphology was observed in the BCCAO/R+BtD group (Figure 6B), with preserved cell structure and intact nuclei, indicating protection against ischemic damage. In contrast, the BCCAO/R+veh group (Figure 6C) showed pronounced histopathological alterations. Purkinje cells were pyknotic, with shrunken and darkly stained nuclei, consistent with advanced nuclear condensation and cell death. Additional features included karyorrhexis and interstitial edema, indicating significant damage due to ischemia/reperfusion.

Treatment with BtD extract (Figure 6D) and BCCAO/R+silymarin (Figure 6E) mitigated these effects. Both groups showed reduced numbers of pyknotic cells and preservation of Purkinje cell morphology, suggesting that the treatments alleviated ischemia-induced cerebellar injury.

## 3. Discussion

This study investigated the neuroprotective potential of a medium polarity root BtD extract from *Bouvardia ternifolia* in a bilateral common carotid artery occlusion and reperfusion (BCCAO/R) model in Sprague Dawley rats. The results demonstrate that BtD effectively attenuates oxidative stress, modulates pro-apoptotic signaling, and preserves neuronal architecture in key brain regions vulnerable to ischemic damage, including the cortex, striatum, hippocampus, and cerebellum.

Oxidative stress, defined by an imbalance between the generation of reactive oxygen species (ROS) and antioxidant defenses, plays a central role in ischemia-induced neuronal injury [29]. In the present study, BCCAO/R significantly increased ROS and lipid peroxidation (LPO) levels, while BtD treatment led to a marked reduction in both markers. These effects suggest potent antioxidant properties of the extract. The extract’s efficacy was further corroborated by changes in antioxidant indicators such as reduced glutathione (GSH) and superoxide dismutase (SOD). The decrease in GSH levels likely reflects its consumption during oxidative stress, while modulation of SOD activity indicates an adaptive enzymatic response. These findings are consistent with the role of ROS in mitochondrial lipid peroxidation, endothelial dysfunction, and the oxidative modification of nucleic acids and proteins [30,31,32].

Additionally, BtD significantly downregulated p53 protein expression. The tumour suppressor p53 is a key mediator of cell cycle arrest and apoptosis, and its overexpression following ischemic injury has been implicated in exacerbating neuronal loss [33,34,35]. The ability of BtD to restore p53 levels to near baseline suggests a protective mechanism that suppresses apoptotic signalling and may promote neuronal survival.

**Figure 7:**
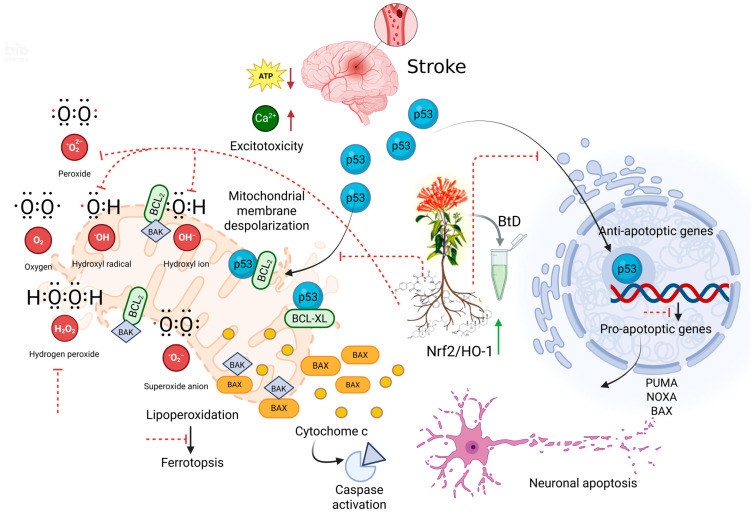
Proposed mechanism by which major phytoconstituents of *Bouvardia ternifolia* (BtD) extract modulate p53 trafficking and mitochondrial-to-nuclear signaling in cerebral ischemia–reperfusion injury. Ischemia–reperfusion induces ROS generation and mitochondrial dysfunction, which triggers p53 stabilization and translocation to both mitochondria and the nucleus, leading to neuronal apoptosis, where it directly binds to, and inactivates, the antiapoptotic proteins B-cell lymphoma-2 (BCL-2) and B-cell lymphoma extra-large (BCL-xL), leading to B-cell lymphoma-2 associated X-protein (BAX) and B-cell lymphoma-2 antagonist/killer (BAK) oligomerization. The pores in the mitochondrial outer membrane trigger cytochrome C release into the cytosol, unleashing caspase cascade and neuronal death. Moreover, p53 also localizes into the nucleus, where it binds to the p53-responsive elements located at target gene promoters, where it initiates its transcriptional apoptotic program, activating proapoptotic genes such as BAX, p53 upregulated modulator of apoptosis (PUMA), NADPH oxidase activator (NOXA), and BID, or inhibiting the expression of BCL-2 and myeloid cell leukemia 1 (MCL-1) antiapoptotic genes. BtD compounds act by reducing ROS and lipid peroxidation, activating the Nrf2/HO-1 axis, preserving mitochondrial membrane potential, inhibiting kinase (p38/JNK) activation, and thus limiting p53 activation and translocation. These combined actions result in decreased p53-mediated apoptotic signaling and increased neuronal survival. Created in BioRender. Zapata, M. (2025) https://BioRender.com/a86w1px.

The modulation of p53 by *Bouvardia ternifolia* BtD extract may result from both direct molecular interactions and indirect antioxidant-mediated signaling (Figure 7). The identified phytochemical profile including ternifolial, rubiyunnanin H, α-tocopherol, squalene, lup-20(29)-en-3-ol-acetate, 3-carene, and various terpenoids and ketones supports several plausible mechanisms:

1. Indirect regulation via antioxidant and anti-inflammatory activity. Several BtD constituents possess strong free radical–scavenging and lipid peroxidation–inhibiting capacities. α-Tocopherol (vitamin E) is a well-characterized chain-breaking antioxidant that prevents lipid peroxidation and reduces ROS accumulation. Lower ROS levels attenuate p53 gene activation driven by redox-sensitive pathways such as MAPK, JNK, and NF-κB. Squalene similarly decreases oxidative stress by enhancing endogenous antioxidant enzyme activities (SOD, CAT, and GSH-Px), leading to stabilization of mitochondrial membranes and reduced oxidative DNA damage—one of the main activators of p53 signaling. 3-Carene and other monoterpenes have been shown to inhibit inflammatory mediators (TNF-α, COX-2, iNOS), thus indirectly reducing stress signaling that upregulates p53 expression. Together, these effects suggest that BtD restores redox balance, lowering the need for p53-mediated stress responses [36].
2. Transcriptional Crosstalk with the Nrf2/HO-1 Pathway. Many natural antioxidants activate Nrf2, which suppresses oxidative and apoptotic pathways while indirectly modulating p53 [37]. BtD components like α-tocopherol, squalene, and rubiyunnanin H are potential Nrf2 activators. Upon activation, Nrf2 upregulates HO-1, NQO1, and GCLC, reducing ROS-induced p53 activation and apoptosis. Hence, BtD may modulate p53 transcriptionally via Nrf2-dependent antioxidant gene induction, maintaining neuronal viability during ischemia/reperfusion injury. Compounds present in BtD extract could inhibit p53 translocation to the outer mitochondrial membrane, where it directly binds to and inactivates the anti-apoptotic proteins B-cell lymphoma-2 (BCL-2) and extra-large B-cell lymphoma (BCL-xL). This results in oligomerization of B-cell lymphoma-2-associated X protein (BAX) and B-cell lymphoma-2 antagonist/killer (BAK). However, further experiments are required to determine the pathway of p53 inhibition [38].
3. Post-translational Regulation: MDM2–p53 Stability. Polyphenolic and terpenoid compounds can also modulate p53 turnover. By reducing oxidative activation of kinases (p38, JNK), BtD may limit p53 phosphorylation and stabilization. Additionally, activation of PI3K/Akt by certain BtD metabolites could enhance MDM2-mediated ubiquitination and proteasomal degradation of p53, restoring physiological levels [39].

Histological assessments further supported the neuroprotective effects of BtD. In the hippocampus—particularly the CA1 and CA3 subfields and the dentate gyrus (DG)—BtD treatment preserved neuronal integrity, reduced interstitial edema, and prevented nuclear fragmentation. These regions are highly susceptible to ischemia due to their elevated metabolic demands and roles in memory consolidation and [40,41,42]. Similarly, in the cerebellum, BtD preserved the morphology of Purkinje cells, which are particularly vulnerable to oxidative damage due to their high energy requirements and complex dendritic architecture [43].

The extract also provided histological protection in the striatum and primary motor cortex (M1), regions involved in motor coordination and voluntary movement. In untreated ischemic animals, these areas exhibited classic signs of neurodegeneration, including pyknosis, cytoplasmic atrophy, and disrupted cellular arrangement. BtD treatment mitigated these changes, suggesting a possible role in preserving motor function and enhancing recovery [44].

Collectively, these findings support the hypothesis that BtD exerts neuroprotective effects by reducing oxidative stress, modulating redox homeostasis, and inhibiting p53-mediated apoptotic pathways. When compared with silymarin, a well-established antioxidant compound, BtD showed comparable efficacy. The observed effects may be attributed to bioactive components previously identified in the extract, including α-tocopherol, lupeol acetate, squalene, and rubiyunnanin H—compounds known for their antioxidant and anti-inflammatory properties [28,45].

Silymarin has been shown to enhance endogenous antioxidant defenses and reduce lipid peroxidation, nitric oxide (NO), and malondialdehyde (MDA) levels. They increase nuclear factor erythroid 2-related factor 2 (Nrf2) expression and upregulate heme oxygenase-1 (HO-1) and NAD(P)H: quinone oxidoreductase 1 (NQO1). Additionally, they preserve Na^+^–K^+^ ATPase activity and maintain mitochondrial membrane potential, thereby suppressing mitochondrial permeability transition pore (mPTP) opening [46].

Silymarin also upregulate proliferator-activated receptor gamma coactivator 1-alpha (PGC1-α), uncoupling protein 2 (UCP2), and nuclear respiratory factor 1 (NRF1), while reducing the expression of inducible NO synthase (iNOS), cyclooxygenase-2 (COX-2), and myeloperoxidase (MPO). They inhibit key transcription factors, including nuclear factor-kappa B (NF-κB), and prevent IκB-α degradation. Proinflammatory cytokines such as TNF-α, IL-1β, and IL-6 are also attenuated [46].

The compound exhibits anti-apoptotic effects by increasing Bcl-2 expression and decreasing p53, Bax, caspase-3, and caspase-9 levels. Functionally, silymarin improves histopathological outcomes, behavioral performance, and reduces cerebral infarct size, highlighting its neuroprotective potential in ischemic brain injury [46].

In conclusion, this study provides compelling evidence for the neuroprotective potential of *Bouvardia ternifolia* root extract in ischemic brain injury. Further research is warranted to isolate and characterize the active constituents responsible for these effects, elucidate their mechanisms of action, and assess their therapeutic utility in models of chronic neurodegenerative disease.

## 4. Materials and Methods

### 4.1. Plant Material and Extract Preparation

A medium-polarity extract from the root of *Bouvardia ternifolia* (Cav.) Schltdl. (Voucher No. INAH-MOR-2080) was obtained using dichloromethane as the extraction solvent. The extract was subsequently fractionated and purified using thin-layer chromatography (TLC) and open column chromatography. Structural characterization of the major compounds was carried out using one-dimensional (^1^H and ^13^C NMR) and two-dimensional nuclear magnetic resonance (NMR) techniques, including correlated spectroscopy (COSY), heteronuclear single quantum coherence (HSQC), and heteronuclear multiple bond coherence (HMBC), along with gas chromatography, as previously described [28].

BtD extract (5 mg) was analyzed by GC-MS using an Agilent/HP 6890 gas chromatograph (Agilent Technologies, Santa Clara, CA, USA) coupled to a quadrupole mass spectrometer (5973 MSD) equipped with a 5MS-l capillary column (30 m × 0.25 mm i.d., 0.25 μm film thickness). The oven temperature was initially held at 40 °C for 1 min and then increased at 10 °C/min to 280 °C. The inlet temperature was set to 250 °C. Mass spectrometric detection was performed in positive electron impact (EI) mode at 70 eV. Samples (1 μL) were injected using helium as the carrier gas at a flow rate of 1 mL/min. Detection was carried out in selective ion monitoring (SIM) mode, and peaks were identified and quantified based on their target ions. Compound characterization was performed by comparing the obtained mass spectra with the NIST 1.7a library. Relative percentages of each compound were calculated by integrating peak areas using GC ChemStation software (v C.00.01), and the composition was expressed as a percentage of the total peak area [28].

The BtD extract (5.5 g) was fractionated using successive open-column chromatography with silica gel (60, F254, Merck KGaA, Darmstadt, Germany) as the stationary phase. A gradient elution system of n-hexane, ethyl acetate, and methanol with increasing polarity was employed. Fractions of 40 mL each were collected and combined into four subfractions: BtD1, BtD2, BtD3, and BtD4. Subfraction BtD4 (116 mg) was acetylated and further separated into four additional subfractions: BtD4.1, BtD4.2, BtD4.3, and BtD4.4 [28].

Separation was monitored using TLC and HPLC. TLC plates included aluminum sheets coated with silica gel 60 F254 (normal phase, Merck KGaA) and silica gel 60 RP-18 F254S (reverse phase, Merck KGaA). Plates were visualized under an ultraviolet light lamp (UVGL-58, 254–365 nm) and using specific developers.

HPLC analyses were performed on a system consisting of a Waters 2695 chromatographic separation module coupled to a Waters 2996 photodiode array detector, with a 250 × 4 mm Licrosphere^®^ (Merck KGaA, Darmstadt, Germany) 100 RP-18 column (5 μm particle size). The mobile phase was a gradient of water and acetonitrile. Samples were prepared at 400 μg/mL, injected at 10 μL, and analyzed at a flow rate of 0.9 mL/min. Detection of compounds was performed over a wavelength range of 195–600 nm.

The principal compounds identified in the extract were: ternifolial (40 mg), rubiyunnanin H (116 mg), (M) lup-20(29)-en-3-ol-acetate (3β) (25 mg), (M) D, α-tocopherol (60 mg), (R) squalene (20 mg), (M) 1H-inden-1-one, 5-(1,1-dimethyl-ethyl)-2,3-dihydro-3,3-dimethyl-, (M) 2-nonadecanone, (M) s-indaceno-1,7-dione, 2,3,5,6-tetrahydro-3,3,5,5-tetramethyl-, and (M) 3-carene (10 mg).

### 4.2. Animals for Experimentation in Cerebral Ischemia

For the animal studies, 9-week-old male Sprague-Dawley rats with an average weight of 350 ± 50 g were used. The rats were housed individually in cages under controlled conditions, with a room temperature of 25 ± 1 °C, humidity at 50 ± 10%, and a 12 h light/dark cycle. They had unrestricted access to food and water. The experiments adhered strictly to Mexican regulations for the care of experimental animals (NOM-062-ZOO-1999 [47]). The study protocol received approval from the Institutional Research Committee and the Ethics Committee of the Mexican Social Security Institute (IMSS) (Registration number R-2020-1702-033). Rats were numbered in order of arrival, and a random number table was generated in Excel. Rats were assigned to groups without any influence from the researcher. The randomization sequence was recorded and kept confidential until administration. The researcher who administered the treatments did not assess the outcomes; the researchers who measured infarct size, biomarker levels, or behavior were blinded to the group assignment.

### 4.3. Induction of Cerebral Ischemia Through Bilateral Common Carotid Artery Occlusion (BCCAO)

Rats were anesthetized with an intraperitoneal injection of 10% ketamine and 2% xylazine. (a) Each rat was placed in a supine position, and a midline cervical incision was made. The submandibular glands, thyrohyoid (TH) muscles, and sternocleidomastoid (SCM) muscles were carefully exposed. (b) The SCM muscles were retracted laterally to allow visualization and dissection of both common carotid arteries, which were carefully separated from the vagus nerve. Vascular clips were then applied bilaterally near the bifurcation of the external and internal carotid arteries to induce cerebral ischemia. The occlusion was maintained for 60 min to induce ischemia, after which the clips were removed to allow reperfusion. To prevent variability due to hypothermia, rats were maintained in an incubator at 30 °C for 3 h post-reperfusion before being returned to their cages.

Animals were randomly assigned to four experimental groups (*n* = 7 per group):

- Group I (Sham): Healthy control rats subjected to sham surgery without arterial occlusion.
- Group II (BCCAO/R+veh): Rats subjected to bilateral common carotid artery occlusion and 60 min of reperfusion, receiving vehicle treatment.
- Group III (BCCAO/R+silymarin): Rats subjected to BCCAO/R and treated with silymarin at 50 mg/kg [46].
- Group IV (BCCAO/R+BtD): Rats subjected to BCCAO/R and treated with dichloromethane root extract of *Bouvardia ternifolia* (BtD) at 300 mg/kg.

Treatments were administered orally, one hour before ischemia/reperfusion induction and twice daily for six consecutive days after ischemia/reperfusion. On day 6, all animals were euthanized, and their brains were collected for the assessment of oxidative stress biomarkers, p53 protein expression, and histopathological analysis.

The sample size (*n* = 6–7 per group) was determined based on previous studies using similar experimental models, which reported significant effects with comparable group sizes. Additionally, a power analysis was performed prior to the study to ensure adequate statistical power (80%) to detect meaningful differences at a significance level of α = 0.05. This approach was intended to balance robustness of the results with ethical considerations for the use of laboratory animals

### 4.4. Brain and Sample Preparation

Following the completion of the experimental procedures, rats were euthanized with an overdose of sodium pentobarbital (100 mg/kg body weight, intraperitoneally), followed by an intracardiac perfusion with isotonic sodium chloride solution. The brains were rapidly extracted and either snap-frozen at −80 °C for biochemical analysis or fixed in 10% paraformaldehyde for 48 h for subsequent histological evaluation.

### 4.5. Measurement of Oxidative Stress Biomarkers

Half of the brain was homogenized in 2 mL of phosphate buffer (10 mM, pH 7.4) and utilized for all oxidative stress measurements and Western blot analysis.

#### 4.5.1. Determination of Lipid Peroxidation (LPO)

LPO was quantified by measuring liposoluble fluorescence, as previously described by [48]. Briefly, each brain tissue sample was homogenized in 2 mL of phosphate buffer (pH 7.4). A 200 µL aliquot of the homogenate was mixed with 4 mL of a chloroform–methanol solution (2:1, *v*/*v*). The mixture was vortexed for 15 s and then incubated on ice for 30 min to allow phase separation. The chloroform phase was subsequently collected and analyzed using a Perkin-Elmer LS55 fluorometer (PerkinElmer, Waltham, MA, USA) set to excitation and emission wavelengths of 370 nm and 430 nm, respectively. Instrument sensitivity was standardized to 140 fluorescence units using 1 μg/mL quinine sulfate in 0.05 M H_2_SO_4_ as a reference. LPO levels were expressed as relative fluorescence units (RFU) per milligram of protein.

#### 4.5.2. Quantification of Reactive Oxygen Species (ROS)

ROS levels were quantified based on the formation of 2′,7′-dichlorofluorescein (DCF), as described by [48]. Briefly, 2 µL of brain homogenate was mixed with 1948 µL of TRIS-HEPES buffer (18:1) and incubated with 50 µL of 2′,7′-dichlorofluorescein diacetate (DCFH-DA) for 1 h at 37 °C. The reaction was terminated by freezing the samples. Fluorescence intensity was measured using a Perkin-Elmer LS-55 fluorometer (PerkinElmer, Waltham, MA, USA) at excitation and emission wavelengths of 488 nm and 525 nm, respectively. ROS levels were expressed as picomoles of DCF formed per milligram of protein per hour [48].

#### 4.5.3. Determination of Superoxide Dismutase (SOD) Activity

SOD activity was measured using a commercial SOD assay kit (Catalog No. 19160, Sigma-Aldrich, Waltham, MA, USA), following the manufacturer’s instructions. Briefly, 20 µL of brain homogenate, 200 µL of working solution, and 20 µL of dilution buffer were combined in each well. The reaction mixture was incubated at 37 °C for 20 min, and absorbance was measured at 450 nm using a microplate reader. SOD activity was expressed as units per milligram of protein (U/mg), where one unit is defined as the amount of enzyme required to catalyze the conversion of 1 µmol of substrate per minute under standard assay conditions [49].

#### 4.5.4. Determination of Reduced Glutathione (GSH)

To quantify the markers of the redox environment GSH, the Hissin and Hilf procedure was utilized. In summary, the brain was homogenized in 2 mL of 10 mM phosphate buffer at pH 7.4. Homogenates (150 μL) were treated with 30% phosphoric acid and then centrifuged at 10,000× *g* for 15 min. For GSH determination, 30 μL of a 1:10 diluted supernatant with FEDTA (100 mM phosphate and 5 mM EDTA) was mixed with 1.9 mL of FEDTA, followed by reaction with 100 µL of O-phthaldialdehyde. Next, 100 μL of O-phthaldialdehyde was added, and fluorescence was measured using a luminescence spectrophotometer (PerkinElmer, Waltham, MA, USA) at excitation and emission wavelengths of 320 nm and 420 nm, respectively. The results were expressed as moles of GSH per milligram of protein [50].

### 4.6. Protein Quantification

The protein concentration in the homogenates was measured using the Bradford method [51].

### 4.7. Western Blot Analysis of p53

The expression of p53 protein was assessed using Western blot analysis. Briefly, 50 μL of brain homogenate was mixed with 5 μL of protease inhibitor cocktail^®^ (MilliporeSigma, Burlington, MA, USA) in lysis buffer, followed by the addition of 45 μL of 2× Laemmli loading buffer (Bio-Rad, Hercules, CA, USA; Catalog No. 161-0737). Samples were vortexed, incubated in a boiling water bath for 3 min, and then stored at −20 °C until analysis. Protein concentrations were quantified, and 50 μg of total protein (in 3 μL) were loaded onto a 10% SDS-polyacrylamide gel and separated via electrophoresis at 70 V for 120 min. Proteins were then transferred to PVDF membranes using the Trans-Blot Turbo transfer system (Bio-Rad) at 25 V and 2.05 A for 12 min. Membranes were blocked for 1 h at room temperature with constant agitation in PBST (PBS containing 0.05% Tween 20 and 5% low-fat milk Svelty^®^(Nestlé, Vevey, Switzerland) and incubated overnight at 4 °C with the primary antibody diluted 1:1000 in blocking buffer. The primary antibody used was anti-p53 (phospho-Thr155, clone D-9; Santa Cruz Biotechnology, Dallas, TX, USA; sc-377567). After incubation, membranes were washed three times (15 min each) in fresh PBST and then incubated for 1 h at room temperature with gentle agitation with secondary antibodies (goat anti-rabbit-HRP and goat anti-mouse-HRP; Life Technologies, Rockford, IL, USA; Catalog No. 65-6120), each diluted 1:1500. Following incubation, membranes were washed four times with PBST.

Protein bands were visualized using chemiluminescence detection with Luminata™ Forte^®^ substrate (Millipore Sigma, Burlington, MA, USA) and captured on photographic film (JUAMA, Mexico City, Mexico). β-Actin was used as a loading control (Santa Cruz Biotechnology, Dallas, TX, USA); sc-47778, dilution 1:4000). Band optical densities were quantified using ImageJ software (version 1.53t; NIH, Bethesda, MD, USA) and results were expressed as the ratio of target protein to β-actin [52].

### 4.8. Preparation of Brain Sections

Histopathological analysis was performed to assess neuronal damage. Whole brains were fixed in 10% paraformaldehyde in phosphate-buffered saline (PBS) for 48 h. After fixation, tissue samples from the cortex, striatum, hippocampus, and cerebellum were processed through graded alcohols and xylene, then embedded in paraffin. Serial coronal sections (7 µm thick) were obtained using a standard microtome. To ensure representative sampling, every 12th section was collected, and 10 sections per animal were selected using a uniform systematic random sampling method.

To evaluate the potential morphological effects of the extract alone, an additional control group (Sham+BtD) was included, in which sections from the hippocampus and cerebellum were analyzed to confirm that the administration of *Bouvardia ternifolia* extract did not alter normal neuronal morphology.

#### Toluidine Blue Staining

For histological staining, a crystal violet solution was prepared using 0.1 g of crystal violet powder (Sigma-Aldrich), 100 mL of distilled water, and 0.7 mL of acetic acid. The solution was filtered through standard filter paper and stored at room temperature until use.

Paraffin-embedded brain tissue sections were subjected to the following sequential staining protocol:

1. Xylene for 5 min;
2. 100% ethanol for 5 s;
3. 96% ethanol for 5 s;
4. Distilled water for 5 s;
5. Crystal violet solution (diluted 30–40% with distilled water) for 5 s;
6. 96% ethanol for 5 s;
7. 100% ethanol for 5 s;
8. Xylene for 5 min.

After staining, the sections were mounted with synthetic resin and cover-slipped for microscopic examination [53].

### 4.9. Statistical Analysis

Statistical analysis was performed using GraphPad^®^ Prism version 9 (GraphPad Software Inc., La Jolla, CA, USA). Data are expressed as the mean ± standard error of the mean (SEM). Comparisons between groups were made using one-way analysis of variance (ANOVA), followed by Dunnett’s post hoc test to compare treatment groups against the BCCAO/R group. Statistical significance was defined as follows: *p* < 0.05 (*), *p* < 0.01 (**), *p* < 0.001 (***), and *p* < 0.0001 (****). Results not reaching statistical significance are denoted as “ns” (not significant).

## 5. Conclusions

The results of this study demonstrated that the root extract of *Bouvardia ternifolia* (BtD) possessed significant neuroprotective potential against brain injury induced by ischemia and reperfusion (BCCAO/R) in Sprague Dawley rats. BtD markedly reduced oxidative stress, as evidenced by decreased levels of reactive oxygen species (ROS) and lipid peroxidation (LPO), while promoting the maintenance of endogenous antioxidant systems such as reduced glutathione (GSH) and superoxide dismutase (SOD).

Furthermore, the extract modulated the p53-dependent pro-apoptotic pathway, reducing its overexpression and thereby limiting neuronal death mechanisms. Histological analysis showed that BtD preserved neuronal structural integrity in brain regions highly vulnerable to ischemic injury, including the hippocampus, cortex, striatum, and cerebellum, preventing typical degenerative alterations such as nuclear fragmentation and cytoplasmic atrophy.

When compared with silymarin, BtD exhibited comparable efficacy, suggesting that its effects were related to the presence of bioactive compounds such as α-tocopherol, lupeol acetate, squalene, and rubiyunnanin H. Collectively, these findings indicated that *Bouvardia ternifolia* represented a promising source of therapeutic agents against ischemic brain injury. Future studies were recommended to isolate and characterize its active constituents and to evaluate their potential in models of chronic neurodegenerative diseases.

Based on the present findings, BtD demonstrates robust antioxidant, anti-apoptotic, and neuroprotective effects in a preclinical model of cerebral ischemia/reperfusion. These effects, particularly the downregulation of p53 and preservation of neuronal integrity, suggest that BtD or its bioactive constituents (e.g., α-tocopherol, squalene, lupeol acetate, rubiyunnanin H) could serve as promising candidates for nutraceutical or pharmaceutical development aimed at mitigating ischemic brain injury.

## 6. Limitations of the Study

In the present study, the focus was on biochemical and histopathological endpoints to establish the neuroprotective and antioxidant effects of *Bouvardia ternifolia* (BtD). However, we fully recognize the importance of functional outcomes for translational relevance. Future studies are planned to include neurobehavioral and cognitive assessments, such as memory tests (Morris water maze, novel object recognition) and motor function evaluations (rotarod, open field), to complement the biochemical and morphological findings and provide a comprehensive evaluation of BtD’s neuroprotective efficacy.

While our current study focused on the integrated biological effect of the whole extract, we fully agree that future work should include fractionation and combinatorial assays to elucidate the relative and synergistic contributions of individual components. This approach would help clarify whether the observed neuroprotection arises from a dominant compound or from the interaction of multiple bioactive molecules.

The current study primarily aimed to provide qualitative histological evidence supporting the biochemical findings. However, we agree that incorporating morphometric or stereological analyses—such as quantification of normal neurons, neuronal density, or infarct volume—would strengthen the objectivity and robustness of the results. These quantitative assessments are being considered for future studies to complement histological observations and provide a more comprehensive evaluation of neuroprotection.

## Data Availability

Data is contained in the paper.
